# Virtual Visits in Pediatrics—Readiness, Barriers and Perceptions Among Healthcare Professionals: A Cross-Sectional Survey

**DOI:** 10.3390/children13010031

**Published:** 2025-12-25

**Authors:** Isabel Castro Garrido, Tregony Simoneau, Jonathan M. Gaffin, Miren Ibarzabal Arregi, María Gimeno Castillo, Claudia Maria Chaverri Reparaz, Alejandro Fernandez-Montero, Laura Moreno-Galarraga

**Affiliations:** 1 Department of Pediatrics, Hospital Universitario de Navarra, HUN, 31008 Pamplona, Spain; 2 Division of Pulmonary Medicine, Boston Children’s Hospital, Harvard Medical School, Boston, MA 02115, USA; tregony.simoneau@childrens.harvard.edu (T.S.);; 3 Instituto de Investigación Sanitaria de Navarra (IdiSNA), Health Research Institute of Navarra, 31006 Pamplona, Spain; 4 Department of Occupational Medicine, University of Navarra, UNAV, 31008 Pamplona, Spain; 5 Health Science Faculty, Medicine, Public University of Navarra, UPNA, 31006 Pamplona, Spain

**Keywords:** virtual visits, pediatrics, telemedicine, healthcare professionals, needs assessment, CFIR

## Abstract

**Highlights:**

**What are the main findings?**

- Pediatric healthcare professionals show a high interest in developing virtual visits (VVs), as an alternative and complementary option to in-person care, and they considered them especially useful for follow-up appointments, specialist consultations, and care of older children and adolescents.
- Primary care pediatricians have fewer technical resources and lower interest, while hospital-based pediatricians report better equipment and greater interest in VVs. In both settings, interest decreases as professionals’ age increases, and older clinicians report more perceived barriers, especially low confidence in handling computers or digital tools.

**What are the implications of the main findings?**

- Successful implementation requires improving technical infrastructures and providing specific training and preparation, especially for older professionals.
- Primary care professionals will additionally require stronger technical support to ensure equitable and effective implementation.

**Abstract:**

**Background/Objectives**: This study explores the perceptions, experiences, and expectations of pediatric healthcare professionals regarding the implementation of virtual visits (VVs) in routine pediatric practice. **Methods**: Using the Consolidated Framework for Implementation Research (CFIR) to analyze individual, organizational, and contextual factors influencing the adoption of pediatric virtual visits, we conducted a descriptive cross-sectional survey distributed nationwide among pediatricians, pediatric nurses, and residents. **Results**: A total of 308 Spanish healthcare professionals correctly completed the REDCap survey and were included in the analysis. The mean age was 44.3 years, and respondents represented both hospital-based (55.8%) and primary care professionals (44.2%). Overall, 74.8% had previous experience with telephone consultations, while only 11% had performed virtual visits. Most professionals believed VVs could be useful in primary care (81.3%) and hospital out-patient settings (73.9%), especially for follow-up appointments, communication of test results, and chronic-care monitoring. VVs were perceived as more appropriate for older children and adolescents than for infants. Major concerns included poor internet connection (52.6%), and data security (37.4%); however, a particularly relevant finding was the low confidence in using digital tools, particularly among older professionals. Comparative analyses by age and workplace setting identified differences in interest, perceived barriers, and access to technical resources. Hospital-based clinicians reported greater interest in adopting VVs and better access to technological resources compared with primary care professionals. The professionals’ age was inversely associated with interest in VVs. Notably, 72.6% of respondents expressed interest in receiving specific VV training, and nearly 90% believed virtual visits should be offered in their workplace. **Conclusions**: These findings show a high overall acceptance of VVs but also underline persistent barriers related to infrastructure, digital literacy, and clinical applicability in younger children. Addressing these obstacles through training, improved equipment, and clear clinical protocols will be essential for the successful implementation of pediatric VV programs.

## 1. Introduction

Pediatric healthcare is gradually incorporating new digital tools, and virtual visits (VVs) have emerged as one of the most promising resources to complement traditional face-to-face care. Over the last decade, technological advances and the widespread use of connected devices have increased interest in these new models of clinical care [1].

Telemedicine was first defined in 1998 by the World Health Organization as “the delivery of healthcare services by all healthcare professionals using information and communication technologies for the exchange of valid information for diagnosis, treatment and prevention of disease and injuries all in the interests of advancing the health of individuals and their communities” [2]. Telemedicine has been examined for several decades since then, with a peak at the COVID-19 pandemic, when a real challenge was set up and professionals were compelled to adopt remote care models at a pace never seen before. Virtual visits arose from the necessity of maintaining continuity, reducing infection risk, and managing care when in-person visits were restricted. Reports from multiple countries, such as the European survey from Reingold SM et al., documented a notable increase in online services: during the pandemic, daily use of telemedicine by pediatricians jumped dramatically, with phone calls rising from 4% to 52%, video conferencing from 1% to 7%, and use of photos or video clips from 1% to 19% [3].

Telemedicine is increasingly showing its value in pediatric care, not only by improving accessibility but also by demonstrating measurable clinical benefits, such as in the control and management of chronic diseases. For example, a recent systematic review and meta-analysis of 16 randomized trials including 2642 children and adolescents with asthma reported a significant improvement in asthma control with telemedicine interventions (ACT/c-ACT mean difference 0.61, 95% CI 0.32–0.90) [4]. Another systematic review revealed that diagnostic agreement between pediatric tele-dermatologists and in-person dermatologists reached 70–89%, with high satisfaction in patients and providers [5]. Furthermore, there are other studies in which telemedicine interventions were designed to facilitate parent–adolescent communication in type 1 diabetes and significant advantages were observed in adherence and quality of life [6,7].

These new tools not only bridged healthcare issues during lockdowns but also revealed new advantages such as reduced travel burden and school- and work-absenteeism, improved follow-up attendance, improved quality of life, and streamlined triage for low-severity noncritical conditions [8,9]. At the same time, the expansion of VVs highlighted several barriers, such as social and structural inequities in both families’ and professionals’ access to technology and connectivity, emphasizing the need for long-term strategies that support efficient, equitable and sustainable digital care [10,11].

The successful integration of virtual visits into routine clinical practice depends heavily on the acceptance, engagement, and readiness of patients and healthcare professionals. These involve several interdependent factors: clinician training and confidence with technology, clear guidance on appropriate clinical use, secure and reliable platforms, and efforts to reduce digital-access inequities. Strategies shown to improve acceptance include practice-level training, integration of telehealth into scheduling, and technical support [12,13,14].

Before implementing a virtual visit program, it is essential to understand the perspectives of both families and healthcare professionals, including their expectations, suggestions, concerns, and perceived barriers. The Consolidated Framework for Implementation Research (CFIR), a comprehensive implementation framework that integrates individual, organizational, and contextual determinants, can guide the assessment of factors that could influence the implementation of a VV program. We designed a two-part preliminary evaluation to ensure that the implementation would be aligned with the needs and expectations of both families and healthcare professionals. The first component—already completed and published—focused on the parents, caregivers, and pediatric patients. In that study, published in *Anales de Pediatría*, we evaluated the interest, expectations, and perceived usefulness and barriers of VV [15]. The survey included 426 participants (316 caregivers and 110 adolescents). Interestingly, although we initially expected concerns about medical data security to be the major barrier, families reported a different primary limitation: the fear of losing the VV-call and, consequently, missing the appointment (35.6% in caregivers; 31.7% in adolescents), with confidentiality concerns ranking much lower (10% in both groups). Caregivers emphasized advantages, such as avoiding missed work, while adolescents highlighted environmental benefits including reduced travel and pollution. Acceptance was very high in both groups: 87.1% of caregivers and 88.6% of adolescents indicated that VV should be offered in their hospital, as long as they remain a voluntary and complementary option to in-person care. Further opinions and suggestions are detailed in the full published article [15].

Here, we present the second component of our project, centered on the perspectives of pediatric healthcare professionals. CFIR provided a structured approach to identifying barriers and facilitators across multiple domains, including individual beliefs and attitudes, organizational readiness, external factors, and key characteristics of the intervention itself. Using this framework allowed us to systematically examine professionals’ concerns, expectations, and perceived needs, and to better understand the elements that may affect successful integration of virtual visits into routine pediatric care. Physicians, residents, and nurses play a key role in determining whether a new model of care becomes feasible, sustainable, and effective. Their perceptions—including perceived usefulness, anticipated barriers, training needs, and concerns—must be thoroughly understood before large-scale implementation. Evidence gathered during and after the COVID-19 pandemic has shown that these factors vary across clinical contexts but consistently influence whether telemedicine becomes a sustainable component. From a system-level perspective, broad adoption also requires organizational support, appropriate technological infrastructure, and clear clinical pathways that allow virtual care to complement traditional services [14,16]. Understanding these perspectives is a fundamental step in the implementation process.

This study aims to examine the experiences, barriers, opinions, needs and expectations of pediatric healthcare professionals regarding the implementation of a pediatric Virtual Visit program.

## 2. Materials and Methods

We conducted a descriptive cross-sectional study using an online survey aimed at healthcare professionals involved in the care of pediatric patients.

The questionnaire was developed by the research team following the Consolidated Framework for Implementation Research (CFIR) and consisted of more than 90 items distributed across several domains, including professional and workplace characteristics, availability of technical resources, previous experience with telemedicine, perceived usefulness and clinical applicability of virtual visits, perceived barriers and concerns, training needs, and interest in implementation. The survey included different response formats, such as dichotomous (yes/no) questions, multiple-choice items, graded scales (0–10 and 0–100), and free-text responses.

In addition to the professionals from our institution (Hospital Universitario de Navarra), participation was extended to a wide range of pediatric healthcare providers across Spain. Eligible participants included the following: primary care and hospital-based pediatricians, primary care and hospital pediatric nurses, pediatric residents (MIR), and nursing residents specializing in pediatrics (EIR). The only inclusion criteria were being a pediatric healthcare professional currently working in Spain and providing informed consent to participate in the study. There were no exclusionary criteria.

The survey was distributed by institutional email and through QR codes to facilitate broad dissemination. QR codes were shared within professional networks of pediatric staff within the hospital, pediatric and nursing groups in Navarra, and personal and professional mailing list and group chats from other autonomous communities in Spain.

Responses were collected anonymously. Data were collected and managed using REDCap electronic data capture tools, hosted at SEPAR, and the REDCap survey remained open for 12 months. The full REDCap questionnaire is provided as Supplementary Materials.

In the statistical analysis, quantitative variables were described using means and standard deviations, while qualitative variables were presented as percentages. Given the large sample size, parametric tests were applied assuming approximate normality. To examine the association between age and quantitative variables, a simple linear regression model was applied. Age was also categorized into four groups, and chi-square tests were used to assess the association between age categories and qualitative variables. To compare hospital-based versus primary care professionals, chi-square tests were used for qualitative variables, and Student’s t-tests for quantitative variables. Finally, a multiple linear regression model was performed to adjust the analyses for age. All analyses were two-tailed tests, and statistical significance was set at *p* < 0.05. The statistical analysis was performed using Stata version 16.0.

The study was conducted in accordance with the ethical principles of the Declaration of Helsinki and applicable regulatory requirements. The study adhered to institutional ethical principles and complied with data protection regulations. Participation was voluntary and anonymous. Approval was obtained from the HUN Hospital Management and from the local ethics committee (Navarra: CEIN reference PI_2023/55/Protocol version 3.1 of 8 June 2023).

## 3. Results

A total of 339 participants accessed the REDCap survey. Eleven were excluded for not having completed the informed consent and six for not being pediatric healthcare professionals. Only 12 questionnaires (3.5%) were not fully completed and were therefore excluded from the analysis. The REDCap survey was programmed to require completion of all items before submission, automatically highlighting missing responses and redirecting participants to unanswered questions. As a result, given the small proportion of incomplete questionnaires, only fully completed surveys were analyzed, and no data imputation procedures were performed. Finally, 310 healthcare workers were included in the study.

### 3.1. Characteristics of Study Population and Previous Experience with Telemedicine

The mean age was 44.35 years (SD: 12.49), with a range between 22 and 68 years old. All the participants were Spanish pediatric health professionals, most of them hospital-based pediatricians (40.18%), followed by primary care pediatricians (22.09%), primary care nurses (14.42%), and pediatricians in residence (7.67%). Half of the sample (50.82%) were based in Navarra, whereas the other half were from Catalonia (14.91%), Basque Country (11.45%), Andalusia (9.72%), Madrid (5.95%) and other Spanish communities (7.15%).

Regarding professionals’ previous experience, 21.29% did not have background experience with any type of telemedicine, 74.84% reported previous experience with telephonic-visits (audio–consultation) and 10.97% with virtual visits (live audio–video consultation).

### 3.2. Availability of Technical Resources

When asked specifically about the available material at their workplace, half of the participants (54.69%) possessed a computer with a camera and 96.55% had access to internet at their workplace. Meanwhile, 24.45% of the participants believed to have all the necessary material to perform VVs, 39.5% were not sure about it and 36.05% affirmed not to have the material needed.

### 3.3. Perceived Usefulness and Appropriate Clinical Contexts for Virtual Visits

Most of the surveyed professionals believed that VVs could be beneficial in primary care and in hospital-based work (81.29% and 73.87%, respectively), while 7.42% of the health professionals referred that in their opinion VVs are not applicable in any kind of pediatric care. Concerning the specific areas or specific situations in pediatric care, where VVs could be useful, 64.52% of the surveyed participants indicated that VVs could be used for follow-up after a hospitalization, 55.81% after an emergency visit, 39.68% for newborn-postpartum checkups and 79.35% reported that VVs could be used in out-patient clinics. The specialty consultations where VVs were thought to be more useful were Gastroenterology, Endocrinology and Pulmonology (75.2%, 71.0% and 69.7%, respectively), whereas the ones with less utility were Neonatology (40.8%) and Cardiology (39.4%).

Furthermore, most of the health workers specified that the best applications for VVs were reviewing test results (81.61%), follow-up visits (74.52%) and medication monitoring (62.26%). On the other hand, the vast majority considered that VVs are inappropriate for a first consultation (94.14%). When comparing VVs with presential-conventional in-person visits, the most expanded opinion was that virtual visits could be a good alternative option in determined circumstances (77.3%). In contrast, 22.04% suggested that conventional visits are always a better option, whereas only 2 participants (0.66%) reported VVs as the better option.

Health professionals considered VVs more useful as patients become older. Only 39.68% believe them to be useful for infants (<2 years old), whereas 86.45% considered them useful for adolescents (11–15 years old). All these results are presented with a more detailed description in Table 1.

When considering the duration of these visits, almost two-thirds of the participants considered that the optimal duration should be between 5 and 15 min, one-quarter preferred between 16 and 30 min, and 5% chose less than 5 min.

### 3.4. Main Perceived Barriers to Virtual Visits

The study also included questions about the main worries or barriers perceived by professionals. The most frequent one was that poor internet connection could compromise the quality and adequacy of the virtual visit (52.58%), followed by potential information filtration (37.42%) and the lack of confidence in using the computer or the digital tools required to conduct a VV adequately (23.55%). Other reported concerns were not having an appropriate amount of quality time dedicated to the patient without interruptions; not being able to conduct a complete examination, including physical features, or losing critical information, such as non-verbal communication.

### 3.5. Training Needs and Interest in Implementation of Virtual Visits

When offered the possibility of receiving specific VV training, the results indicate that 72.58% of the professionals would be interested. Almost 90% of them maintained that their workplace should offer this training.

Lastly, items assessing participants’ interest in VVs were included. On a scale from 0 to 10 (defining 0 as least interested and 10 as most interested), professionals rated the interest in their healthcare center providing the option of VVs with a mean score of 7.55 (SD 2.41). On the same 0–10 scale, participants reported a mean interest of 7.14 (SD 2.88) in personally offering virtual visits to their own patients.

We also conducted an analysis examining these responses according to the professional’s age and workplace setting (hospital vs. primary care) to determine whether opinions about virtual visits were associated with these factors.

**Figure 1:**
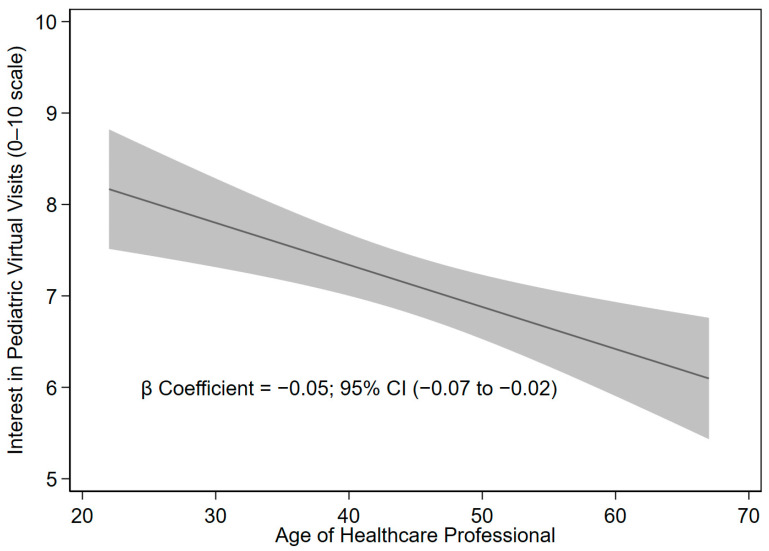
Significant association between grade of interest in pediatric virtual visits (scale from 0 to 10) and age of healthcare professionals (years old). The solid line shows the estimated linear association between age and interest in pediatric virtual visits and the grey bands indicate the 95% confidence interval.

We analyzed the effect of age on variables related to interest in VVs using lineal regression models. Figure 1 illustrates how as the health professional becomes older (represented in the figure on the horizontal axis), interest in virtual visit decreases (represented in the vertical axis on a scale from 0 to 10). (*p* < 0.001, beta coefficient −0.05, SD 0.01). For each additional year of age, the professional’s level of interest decreased by 0.05 points.

**Figure 2:**
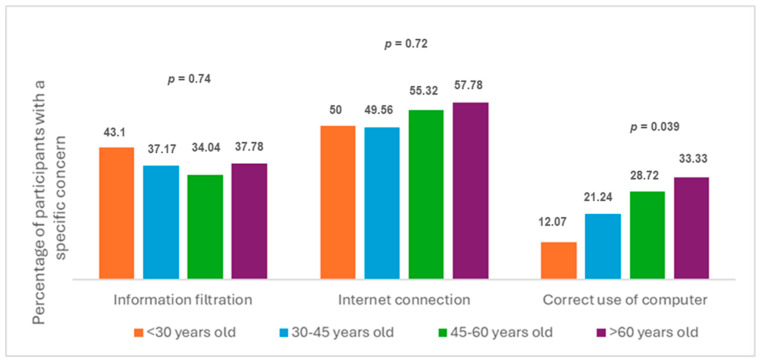
Comparative analysis of the referred key barriers to virtual visits among healthcare professionals across age groups.

To assess whether the reported barriers or concerns were related to the professionals’ age, we categorized age into four groups and examined whether differences existed between extreme age-categories. The sample was divided into four groups: <30 years old (y/o); 30–45 y/o; 45–60 y/o and >60 y/o. When each potential barrier was evaluated independently, we found no significant differences regarding the reported concern of poor internet connection (*p* = 0.72) or the concern of filtration of personal medical information (*p* = 0.74), but we did find significant differences in the fear of not knowing how to use the computer (*p* = 0.039). Overall, older professionals reported a greater number of fears and perceived barriers. The more frequently expressed concern was about not feeling confident in handling the technology or the computer equipment required to conduct a VV. Results are detailed in the bar diagram (Figure 2).

Finally, potential differences between hospital-based and primary care professionals were examined. We found no significant differences between workplaces in most aspects, such as the preferences of VV duration (*p* = 0.729) or interest in VV training (*p* = 0.713). In terms of available material in their workplace, hospital-based professionals are significantly better equipped than primary care pediatricians. Hospital-based pediatricians exhibited greater interest in both implementing virtual visits in their work center and in personally offering them to their patients (*p* < 0.001 in both cases), both assessed on a 0–10 scale. Given that the mean age of hospital-based professionals was slightly lower than that of primary care professionals, the results were first examined in an unadjusted (crude) analysis and subsequently reassessed using a multivariable model adjusted for age (Table 2). Both analyses yielded consistent results.

## 4. Discussion

This study provides an overview of the perceptions and expectations related to VVs in pediatric healthcare professionals.

In general, pediatricians express a favorable attitude towards virtual visits, particularly for follow-up care and for out-patient situations. These findings are consistent with previous studies, reporting that clinicians tend to accept virtual care more easily in clinical scenarios where physical examination is not essential, such as reviewing test results, monitoring chronic conditions, or conducting follow-up appointments. This aligns with prior studies conducted during the COVID-19 pandemic, which described a rapid increase in clinicians’ acceptance of digital tools, driven by necessity but sustained by perceived benefits in areas where the patients may not be needed in-person [17,18].

Interestingly, the perception that VVs are inappropriate for first consultations reinforces the idea that telemedicine should be used selectively, making active choices whether the patient is a good or bad candidate for virtual care, particularly when establishing new diagnosis, developing the patient–provider relationship, or evaluating complex clinical presentations.

Additionally, VVs were perceived as more appropriate in older children and adolescents, while their usefulness in infants was considered limited. This may be because younger patients may not be able to fully describe their symptoms and therefore physical examination becomes crucial for a correct diagnosis. It could also be since VVs might lessen the amount of missed school and, therefore, be more helpful for students in higher grades. [14,15]. Professionals also highlighted that VVs are less applicable in subspecialties where physical examination is more central, such as neonatology, neurology or when complementary tests may be needed, such as cardiology.

The most frequently reported barriers were concerns about internet connectivity, data security and lack of confidence in managing the required digital tools, especially in older professionals. It is worth highlighting that there is a lower level of interest in virtual visits among older professionals, which may be related to the reduced confidence and more preoccupation over inadequate digital skills. This finding aligns with earlier research linking lower digital literacy and telemedicine acceptance to veterans [18,19] and emphasizes the need for specific structured training programs. Mentorship models, hands-on workshops, and incremental exposure could reduce resistance and increase adoption among older clinicians.

Another relevant finding was the discrepancy between hospital-based and primary care professionals. Hospital-based clinicians reported greater interest in adopting and personally conducting VVs than their primary care counterparts. This may be linked to their more frequent exposure to chronically ill or complex patients, who may benefit from remote monitoring. Conversely, primary care professionals may rely more on physical examination and longitudinal relationship-building, which could explain their more cautious stance, as supported in several previous publications [17,20,21]. Also, this likely reflects the differences in technological infrastructure and prior exposure to digital tools.

Results from our previously conducted survey among caregivers and adolescents showed that more than 95% of respondents reported having both internet access and a mobile device with a camera, suggesting that, in our setting, most families have all the necessary technical means to participate in virtual visits. In contrast, access to appropriate equipment, such as a computer with a camera, was less frequently reported among healthcare professionals, highlighting that digital barriers may be more relevant on the provider side than on the patient side in our population, particularly in primary care settings. These results have several implications for healthcare planning. The relatively low proportion of professionals that reported having adequate material to conduct VV underlines the need for healthcare institutions to ensure technical resources and connectivity and to reduce technological gaps, especially in primary care settings. Training programs tailored to the needs of different groups may help address concerns related to digital competency, as well as developing protocols for virtual care.

Based on our findings, implementation strategies should be flexible and supportive rather than mandatory. Inverse mentoring models, in which younger professionals support older colleagues in the use of digital tools, may be particularly useful. Given that older healthcare professionals in our study reported greater concerns about technology and lower confidence in using digital platforms, it seems essential not to force adoption, to respect professional autonomy, and to clearly present virtual visits as a voluntary option. Initial steps could include conducting virtual visits with the support of a resident or younger colleague and offering short, practical training sessions before broader implementation. Exploring professionals’ perspectives through this survey was an important step toward designing a virtual visit program that also respects clinicians’ needs, preferences, and working rhythms.

As with all survey-based studies, certain limitations should be acknowledged. However, the questionnaire was designed by healthcare professionals, reviewed by hospital management, and tested through several pilot rounds before dissemination, which may have contributed to improved clarity, completeness, and reliability of the collected responses.

To our knowledge, our study is the first investigation that aims to learn about experiences and opinions regarding virtual visits in Spanish pediatric healthcare professionals. It includes a large and diverse sample of pediatric healthcare professionals across multiple regions in Spain, representing different disciplines and levels of experience. However, its cross-sectional design limits causal inference, and the reliance on self-reported data may introduce bias. The online nature of the survey may also have inadvertently selected individuals with greater digital skills. Participation was voluntary, and most of the participants were from Navarra, so the sample may over-represent professionals in this area and with stronger interest in virtual care. However, although approximately half of the participants were based in Navarra, a comparative analysis according to place of residence (Navarra vs. other regions of Spain) did not show statistically significant differences in the main study variables. Moreover, given that the pediatric healthcare system in Spain is relatively homogeneous nationwide, with a similar organization based on primary care and hospital-based services across regions, we believe that this geographic distribution is unlikely to have substantially influenced the overall results.

## 5. Conclusions

In summary, Spanish pediatric healthcare professionals generally recognize the value of virtual visits, especially for follow-up and non-urgent consultations in older children and adolescents. However, concerns about technical limitations and varying levels of digital confidence remain key barriers that must be addressed to ensure successful implementation. Strengthening digital infrastructure, providing tailored training, and defining clear clinical pathways will be essential.

Overall, these findings offer practical guidance for institutions to design pediatric virtual visit programs that align with the expectations and needs of both clinicians and families. Based on our findings, several practical recommendations can be proposed for the implementation of pediatric virtual visit programs. First, virtual visits should be offered as a voluntary and complementary option to face-to-face care, respecting clinicians’ preferences and individual readiness. Adequate preparation and prior training are essential, together with ensuring that appropriate technical resources are available and that all professionals have access to the necessary equipment before implementation. Virtual visits seem to be particularly well suited for follow-up of chronic patients and for older children and adolescents, whereas their usefulness appears more limited for first consultations and for younger children. Taking these factors into account may facilitate a more effective, acceptable, and sustainable integration of virtual visits into pediatric care.

## Figures and Tables

**Table 1:** Perceived usefulness and appropriate clinical context for virtual visits in different scenarios included in the study.

Variable	*n* (%)
**General usefulness of VVs in Pediatrics**	
Useful in primary care	252 (81.3)
Useful in hospital-based attention	229 (73.9)
Not useful in any case	23 (7.4)
**Context in which VVs are useful**	
First consultation	18 (5.8)
Follow-up visits	231 (74.5)
Test result reviews	253 (81.6)
Medication monitoring	193 (62.2)
**Clinical contexts in which VVs are useful**	
After hospitalization	200 (64.5)
After emergency visit	173 (55.8)
Newborn-postpartum checkups	123 (39.7)
In out-patient clinics	246 (79.3)
**VV usefulness in out-patient clinics**	
Pediatric pulmonology	219 (69.7)
Pediatric cardiology	125 (39.8)
Pediatric endocrinology	223 (71.0)
Pediatric gastroenterology	236 (75.2)
Pediatric neurology	180 (57.3)
Pediatric nephrology	215 (68.5)
Neonatology	128 (40.8)
**Usefulness according to the patients’ age**	
Infants (<2 years old)	123 (39.7)
Preschoolers (2–5 years old)	192 (61.9)
Children (6–10 years old)	258 (83.2)
Adolescents (11–15 years old)	268 (86.5)
Not valuable at any stage of pediatric care	23 (7.4)

**Table 2:** Workplace-based differences in virtual visit resources, training needs and interest (primary care vs. hospital-based pediatric healthcare professionals).

	Primary Care	Hospital-Based	*p*
Participants (*n*, %)	137 (44.2%)	173 (55.8%)	
Age years (mean, SD)	46 (±11)	43 (±12)	0.032
**Technical Resources Available** Computer with camera (%)	46.32	62.79	<0.001
Internet connection (%)	94.12	98.26	0.052
All required equipment for VV (%)	9.26	28.49	0.012
**Previous telemedicine experience (%)**	71.53	84.39	0.006
**Preferred virtual visit duration**			
<5 min (%)	5.60	5.56	
5–15 min (%)	64.00	69.75	0.729
15–30 min (%)	28.00	22.22	
>30 min (%)	2.40	2.47	
**Perceived training needs (%)**	71.53	73.41	0.713
**Interest**			
Interest in workplace offering VVs (0–10)	6.96 (±2.7)	8.01 (±2.03)	0.0001
Personal interest in providing VVs (0–10)	6.44 (±3.08)	7.69 (±2.60)	0.0002

VV: virtual visit. SD: standard deviation.

## Data Availability

The data collected for this study are available from the corresponding author upon reasonable request. As the dataset includes anonymized survey responses, access may be provided in accordance with ethical and institutional guidelines.

## Supplementary Materials

The following supporting information can be downloaded at: https://www.mdpi.com/article/10.3390/children13010031/s1, File: The full REDCap questionnaire.
