# Supplementary material for: Virtual Visits in Pediatrics—Readiness, Barriers and Perceptions Among Healthcare Professionals: A Cross-Sectional Survey
Source: Children (Basel). 2025 Dec 25;13(1):31. doi: 10.3390/children13010031 (PMC12839630; doi:10.3390/children13010031)
Supplement: Supplementary file 1 [file children-13-00031-s001.zip › children-4054672-Supplementary.pdf]

Visitas Virtuales en Pediatría (versión para profesionales sanitarios)

Por favor, responda a la siguiente encuesta sobre las Visitas Virtuales en Pediatría.

Muchas gracias

¡HOLA!
Esta encuesta incluye preguntas sobre las "Visitas Virtuales" en Pediatría.
Estás accediendo a la versión para profesionales de la salud.
Las "Visitas Virtuales" son un tipo de visitas clínicas en las cuales, previa cita, un profesional sanitario se pone en contacto con el paciente y su familia a través de una plataforma digital tipo "zoom" (con audio y con video). Existen muchos modelos diferentes de visitas virtuales clínicas.

Queremos saber tu opinión y sugerencias sobre este tipo de visitas.

Los resultados y sugerencias de esta encuesta podrán ser utilizados en publicaciones científicas. La encuesta es voluntaria y anónima.

Responder te tomará unos 5 minutos. Por favor responde lo más honestamente posible. No hay respuestas correctas o incorrectas. Es importante que respondas a todas las preguntas. Si no sabes cómo responder, marca la mejor respuesta de entre las opciones presentadas.

¡Muchas gracias por tu ayuda y por tu tiempo!

Dra. Laura Moreno-Galarraga
Equipo Neumología Infantil - Pediatría
Hospital Universitario de Navarra HUN

Entiendo el objetivo de esta encuesta y quiero participar:

- ◯ Sí; quiero completar la encuesta
- ◯ No; No quiero continuar

Las primeras preguntas incluyen información general sobre el profesional sanitario que rellena la encuesta:

¿En qué campo trabajas actualmente?

- ◯ Soy pediatra de atención primaria
- ◯ Soy pediatra de atención hospitalaria (Trabajo habitualmente en consultas)
- ◯ Soy pediatra de atención hospitalaria (No trabajo habitualmente en consultas)
- ◯ Soy residente MIR
- ◯ Soy residente EIR
- ◯ Soy personal de enfermería de atención primaria
- ◯ Soy personal de enfermería de atención hospitalaria
- ◯ Otro profesional sanitario
- ◯ NO soy profesional sanitario

Define por favor tu especialidad y lugar de trabajo habitual

____

¿Trabajas actualmente en Navarra?

- ◯ Sí
- ◯ No

¿En que Comunidad Autónoma trabajas?

____

¿Qué edad tienes?

____
(Indique su edad en años; (mínimo 18))

¿Dispones de un ordenador con cámara en tu lugar de trabajo ?

◯ No
◯ Sí

¿Dispones de conexión a internet en tu lugar de trabajo?

◯ No
◯ Sí

¿Dispones de lo necesario para hacer visitas virtuales en tu lugar de trabajo?
Como autorización de tu centro, o acceso a una plataforma de videoconferencias segura o con conexión desde la propia historia clínica...

◯ No
◯ Sí
◯ No lo sé

Las siguientes preguntas son sobre tu opinión de las Visitas Virtuales en pediatría :

Respecto a tu experiencia con las Visitas Virtuales marca las opciones correctas:
(Puede marcar más de una opción)

☐ No he realizado nunca una consulta telefónica, ni visita virtual o video-conferencia con un paciente
☐ He realizado llamadas de teléfono a mis pacientes (audio)
☐ He realizado Video-llamadas con mis pacientes (audio + video)

Respecto a la utilidad de las visitas virtuales, marca las respuestas que considere correctas: " Creo que las visitas virtuales en pediatría.....
(Puede marcar más de una opción)

☐ podrían ser útiles en Atención Primaria (Centro de Salud)
☐ podrían ser útiles en Atención Hospitalaria
☐ no son utiles en ningún tipo de asistencia pediátrica

Creo que, a NIVEL HOSPITALARIO, las Visitas Virtuales en pediatría, podrían ser útiles:
(Puede marcar más de una respuesta)

☐ En el seguimiento tras un ingreso en el hospital
☐ En el seguimiento tras una visita a urgencias
☐ En el seguimiento de un lactante, tras el nacimiento
☐ En el seguimiento en las CONSULTAS DE ESPECIALIDAD
☐ En ninguna situación

Las últimas preguntas son sobre el uso de las Visitas Virtuales en atención hospitalaria, para su uso en las CONSULTAS DE PEDIATRIA

¿En qué especialidades pediátricas consideras que las Visitas Virtuales podrían ser de utilidad?
(Puede marcar más de una respuesta)

☐ En neumología infantil
☐ En cardiología infantil
☐ En endocrinología infantil
☐ En nefrología infantil
☐ En digestivo infantil
☐ En neurología infantil
☐ En consulta de Neonatología
☐ En otras consultas
☐ En ninguna especialidad pediátrica

Respecto a en que momentos o situaciones se podrían utilizar las Visitas Virtuales para una consulta de especialidad, marque las respuestas que considere correctas: "Creo que las Visitas Virtuales en pediatría podrían ser útiles....
(Puede marcar más de una opción)

☐ Como una primera visita con el especialista
☐ Como una visita de seguimiento con el especialista
☐ Para informar sobre el resultado de pruebas complementarias
☐ Para valorar la evolución de una enfermedad
☐ Para valorar el efecto de una nueva medicación
☐ No son de utilidad en ninguna de estas situaciones

Respecto a en que grupos de edad, considera que las Visitas Virtuales pueden ser útiles, marque las respuestas correctas: "Creo que las Visitas Virtuales en pediatría podrían ser útiles....
(Puede marcar más de una opción)

☐ en lactantes menores de un año
☐ entre los 2-5 años
☐ entre los 6-10 años
☐ entre los 11-15 años
☐ Considero que las visitas virtuales no son útiles en la edad pediátrica

---

Respecto a la duración media de las Visitas Virtuales, Cuánto crees que debería durar una consulta de este tipo:

◯ menos de 5 minutos
◯ 5-15 minutos
◯ 16-30 minutos
◯ más de 30 minutos

Comparando una Visita Virtual con una Visita Presencial tradicional, marque la opción que considere más correcta.

◯ Siempre es mejor una Visita Presencial
◯ Siempre es mejor una Visita Virtual
◯ En algunas situaciones, una visita virtual puede ser una buena alternativa a una visita presencial.

Marca de entre las siguientes opciones, los puntos que te preocupan sobre la instauración de las visitas virtuales: Me preocupa ...

☐ que se filtren datos médicos o personales de mis pacientes
☐ que la conexión a internet no sea estable y la calidad de la llamada sea mala
☐ que no sepa manejar correctamente el ordenador
☐ otras preocupaciones
☐ No tengo preocupaciones respecto a las visitas virtuales

Si quieres, puedes contarnos qué te preocupa de las Visitas Virtuales: __________________________________

Estarías interesado en formación específica sobre el uso de estas visitas virtuales

☐ si ☐ no

Respecto al uso de las Visitas Virtuales, para las consultas de especialidad: En que medida te parece interesante que exista la posibilidad de hacer este tipo de visitas en tu lugar de trabajo

Nada interesante — Muy interesante

(Place a mark on the scale above)

Las últimas preguntas:
¿Cree que es una buena opción que tu centro ofrezca la posibilidad de hacer Visitas Virtuales para el seguimiento de algunas consultas de pediatría?

◯ Sí
◯ No

En una escala del 0 al 10, En que medida valorarías como una buena opción que tu centro de trabajo ofreciera la posibilidad de hacer visitas virtuales

____
((siendo 0= poco interesante, y 10= muy buena opción))

En una escala del 0 al 10, En qué medida TÚ estarías interesado en poder ofrecer a tus pacientes la posibilidad de hacer visitas virtuales

____
((siendo 0= poco interesante, y 10= muy buena opción))
